# MicroRNA-939 restricts Hepatitis B virus by targeting Jmjd3-mediated and C/EBPα-coordinated chromatin remodeling

**DOI:** 10.1038/srep35974

**Published:** 2016-10-25

**Authors:** Cuncun Chen, Min Wu, Wen Zhang, Wei Lu, Min Zhang, Zhanqing Zhang, Xiaonan Zhang, Zhenghong Yuan

**Affiliations:** 1 Institute of Medical Microbiology and Biomedical Sciences, Shanghai Medical College of Fudan University, Shanghai, China; 2 Research Units, Shanghai Public Health Clinical Center, Fudan University, Shanghai, China; 3 Key Laboratory of Medical Molecular Virology, Ministry of Education and Health, Shanghai Medical College of Fudan University, Shanghai, China; 4 Department of Hepatology, Shanghai Public Health Clinical Center, Shanghai, China; 5 Department of Clinical Laboratory, Shanghai Public Health Clinical Center, Shanghai, China

## Abstract

Multi-layered mechanisms of virus host interaction exist for chronic hepatitis B virus (HBV) infection, which have been typically manifested at the microRNA level. Our previous study suggested that miRNA-939 (miR-939) may play a potential role in regulating HBV replication. Here we further investigated the mechanism by which miR-939 regulates HBV life cycle. We found that miR-939 inhibited the abundance of viral RNAs without direct miRNA-mRNA base pairing, but via host factors. Expression profiling and functional validation identified Jmjd3 as a target responsible for miR-939 induced anti-HBV effect. Jmjd3 appeared to enhance the transcription efficiency of HBV enhancer II/core promoter (En II) in a C/EBPα-dependent manner. However, the demethylase activity of Jmjd3 was not required in this process. Rather, Jmjd3’s transactivation activity depended on its interaction with C/EBPα. This coordinated action further recruited the Brm containing SWI/SNF chromatin remodeling complex which promoted the transcription of HBV RNAs. Taken together, we propose that the miR-939-Jmjd3 axis perturbs the accessibility of En II promoter to essential nuclear factors (C/EBPα and SWI/SNF complex) therefore leading to compromised viral RNA synthesis and hence restricted viral multiplication.

Hepatitis B virus (HBV) infection remains as one of the most prevalent viral infection in human beings. It is estimated that around two billion people have evidence of past or present infection with HBV and 240 million are chronic carriers of HBsAg. Some regions, including East Asia and sub-Sarahan African etc, have the highest prevalence (>5%) of HBV. Chronic hepatitis B (CHB) infection is associated with a significantly increased risk for the development of cirrhosis, liver failure, and hepatocellular carcinoma (HCC)^1^,^2^. The efficacy of currently available therapies for CHB including interferon α (IFN-α) or nucleot(s)ide analogues are far from being satisfactory^3^. A better understanding of the HBV-host interaction is urgently needed for the discovery of novel targets for therapeutic intervention.

HBV is a member of the hepadnaviridae, a family of small hepatotropic partially double stranded DNA virus. Its genome is approximately 3.2 kb in length and replicates through reverse transcription. After viral entry, the virion DNA is transported into the nucleus and transforms into covalently closed circular DNA (cccDNA) which serves as genome reservoir and template for transcription of four major transcripts, i.e., 3.5 kb pregenomic RNA (pgRNA) and 2.4 kb, 2.1 kb, 0.7 kb RNAs^4^. Transcription of these RNAs is directed by four distinct promoters namely, preS1 promoter, SP I; preS2 promoter, SP II; Enhancer I/X promoter, En I and Enhancer II/core promoter, En II. In particular, En I and En II not only serve as promoters for their cognate 3′ ORFs but also enhance the production of other transcripts. Extensive studies have shown that transcription factors, including the liver-enriched hepatocyte nuclear factor 1, 3 (HNF-1, 3) and CAAT enhancer-binding protein (C/EBP) etc, play a key role in regulating HBV enhancers^5^,^6^.

MicroRNA (miRNA) are endogenous, non-coding RNAs with length of 20 to 25 nucleotides, which typically mediate messenger RNA (mRNA) cleavage and/or translational repression through partial or complete complementarity between the “seed region” of miRNA^7^,^8^ and the 3′ or 5′-untranslated region (UTR) of target transcripts^9^. miRNAs have emerged as key regulators in eukaryotes, influencing various biological processes like development, infection, immunity, and carcinogenesis. Expressions of distinct miRNAs signatures are associated with particular organs and tumor types^10^,^11^.

A growing body of evidence has demonstrated that viral infection can change host miRNA profile, which may affect the virus-host interactions and participate in the viral life cycle and pathogenesis^12^. Indeed, quite a number reports have also supported this concept in HBV infection^13^,^14^. The altered miRNA expression may directly target viral transcripts, or modulate host genes involved in the viral replication machinery. For example, a liver-specific microRNA, miR-122, was found to bind to the highly conserved HBV pregenomic RNA (pgRNA) sequence via base-pairing interactions, thus inhibiting viral gene expression and replication^15^. On the other hand, miR-122 may also inhibit HBV replication indirectly through targeting cyclin G1, thus abrogating the interaction between cyclin G1 and p53 leading to the enhancement of p53-mediated inhibition of HBV replication^16^.

In a previous study, we showed that a plasma microRNA profile consisting of eleven miRNAs can serve as a predictor of early virological response to IFN treatment in CHB patients. In addition, one of these miRNAs, miR-939, regulated the replication of HBV in hepatoma cell lines^17^. In this study, we further investigated the mechanism by which miR-939 regulates HBV life cycle. We found that miR-939 interfered with HBV transcription by targeting the host genes that are important regulators of viral transcription. Furthermore, we identified Jumonji domain containing 3 (Jmjd3) as the target of miR-939, which promoted the formation of transactivation machinery on HBV enhancer II. Unexpectedly, the demethylase activity of Jmjd3 was not required in this process. Instead, we propose that the transcriptional complex composed of Jmjd3 and C/EBPα coordinated recruitment of Brm containing SWI/SNF chromatin remodeling complex to HBV DNA.

## Results

### Suppression of HBV transcription and replication by miR-939

**Figure 1:**
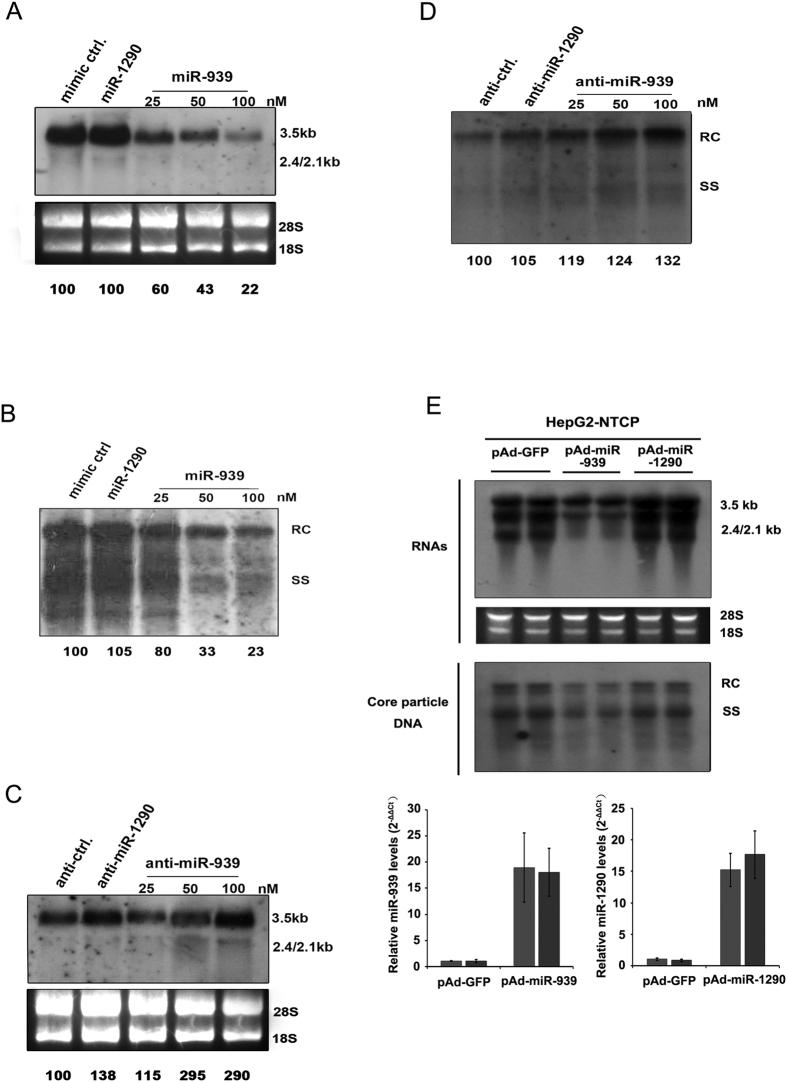
Suppression of HBV transcription and DNA synthesis by miR-939. Indicated concentration of miR-939 mimics (**A,B**) or inhibitors (**C,D**) were co-transfected with pHBV1.3 into Huh7 cells. miR-1290 was used as an unrelated control. (**A,C**) Northern blot analysis of HBV RNAs. 28S and 18S RNA serve as loading control. The location of HBV pregenomic RNA (3.5 kb), preS1/S RNA (2.4 kb) and preS2/S RNA (2.1 kb) are indicated. The densitometry results of HBV RNAs were normalized by loading RNA. (**B,D**) Southern blot analysis of HBV Core particle DNA. The location of HBV relaxed circular DNA (RC) and single stranded DNA (SS) are indicated. Numbers below lanes indicate densitometry results (presented (as %) relative to mimic control or antagomir control). (**E**) HepG2-NTCP cells were transduced with either control or miR-939-expressing adenoviral vectors. 2 days later, cells were infected with HBV in duplicate and then cultured for an additional 7 days. Intracellular viral RNAs and Core particle DNA were analyzed by Northern blotting (top) and Southern blotting (middle). The expression of miR-939 and miR-1290 were analyzed by quantitative RT-PCR (bottom). Full-length gels are presented in Supplementary Figure S7.

Since our previous report indicated that introduction of miR-939 in Huh7 cells can inhibit the secretion of viral antigens^17^, we thus hypothesized that miR-939 served as a negative regulator of HBV replication or transcription. To test this, Huh7 cells were co-transfected with HBV replicative construct pHBV1.3 and different concentrations of miR-939 mimic ranging from 25 to 100 nM. A 40% reduction of HBV pgRNA and other mRNA transcripts was observed at 25 nM of miR-939 and up to 80% at 100 nM, compared with mimic control while miR-1290 mimic did not have a significant effect on HBV transcription (Fig. 1A). HBV replicative intermediates levels were also decreased significantly as determined by Southern blot (Fig. 1B). We next tested whether depletion of endogenous miR-939 can have an opposite effect. After co-transfection of chemically synthesized miR-939 antagomir, an obvious increasing of viral transcripts (Fig. 1C) as well as replicative intermediates (Fig. 1D) were observed compared with antagomir control and miR-1290 antagomir. Furthermore, we confirmed the inhibitory effect of miR-939 on HBV transcription and replication in HBV infection system by using HepG2-NTCP cells. As expected, adenoviral introduction of miR-939 led to a decreased level of both viral transcripts and replication intermediates (Fig. 1E). Together, these results confirmed that miR-939 effectively and specifically inhibited HBV replication at a step prior to viral DNA synthesis.

### Suppression of HBV enhancer II/core promoter (En II) activity by miR-939

**Figure 2:**
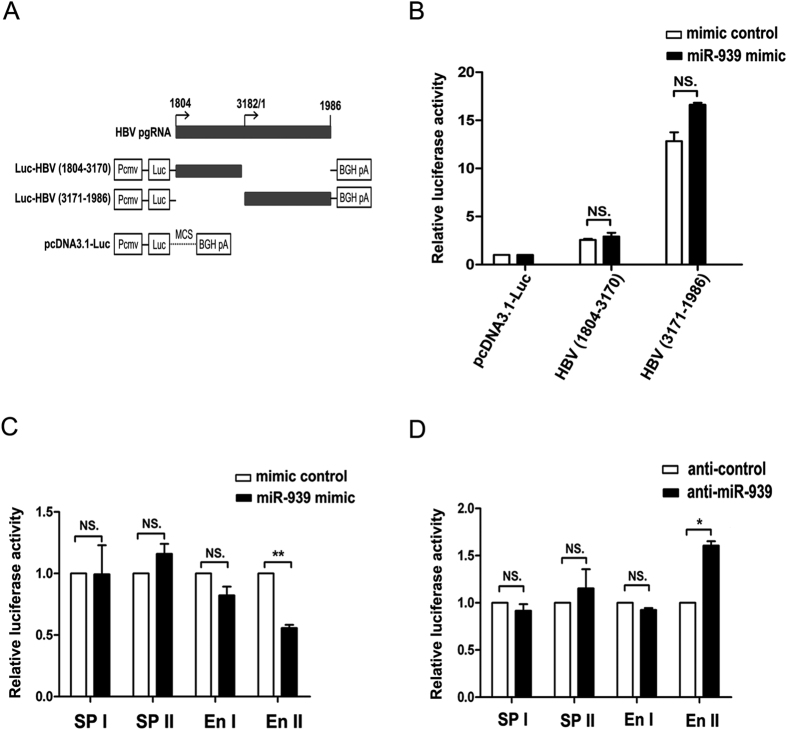
Suppression of HBV enhancer II/core promoter (En II) activity by miR-939. (**A**) Schematic diagram of the chimeric HBV pgRNA luciferase reporter plasmids. (**B**) Huh7 cells were transfected with 0.1 μg indicated luciferase fusion constructs and 100 nM miR-939 mimic or mimic control for 48 h, the luciferase activity was then assessed. Results were normalized according to Renilla luciferase activities and represent the means of data from three independent experiments performed in duplicate. (**C,D**) Specific inhibition of HBV En II transcription activity by miR-939. Four HBV promoter/enhancer reporter plasmids were separately co-transfected with 100 nM miR-939 mimic (**C**) or inhibitor (**D**) into Huh7 cells for 48 h, the luciferase activity was then assessed.

To explore the molecular mechanisms by which miR-939 suppresses HBV transcription, we first tested whether miR-939 can directly target HBV transcripts causing their degradation. However, the complementary sequence of miR-939 seed region was not found in the HBV genomic sequence by bioinformatics software RNA22 analysis. We further utilized a previously established luciferase reporter assay. In brief, the 5′ (1804-3170) and 3′ (3171-1986) half of HBV pgRNA sequence was inserted into the 3′UTR of firefly luciferase open reading frame (pcDNA3.1-Luc)^18^. A direct interaction between miR-939 and HBV RNA would affect the stability of luciferase encoding mRNA (Fig. 2A). We validated this system by utilizing two previously designed siRNAs that direct targeting HBV pgRNA sequences (Fig. S1)^19^. Each of these two reporter plasmids were co-transfected with miR-939 mimic or mimic control into Huh7 cells. It was found that neither of the Luc-HBV reporters was significantly affected by the introduction of miR-939 (Fig. 2B) suggesting a lack of direct base pairing.

We then asked whether the function of HBV promoters/enhancers was affected by miR-939. For this, we first employed a promoter reporter system in which the luciferase gene was driven by four HBV promoter/enhancers. As shown in Fig. 2C, the expression of miR-939 significantly decreased the transcription activity of the HBV En II, which directs pgRNA transcription and also regulates other shorter transcripts, but had no effect on the other three promoters. Conversely, depletion of miR-939 level using antagomir specifically increased the activity of HBV En II (Fig. 2D). To confirm that the antiviral activity miR-939 was dependent on En II transcription, we checked the effects of miR-939 on viral RNA derived from pCMV-HBV, which drives RNA transcription by a CMV promoter. Neither miR-939 mimic nor miR-939 antagomir exhibited an obvious effect on the viral RNA when pCMV-HBV was transfected (Fig. S2). All the above results suggested that miR-939 could not directly target HBV transcripts, but may restrict HBV transcription via the perturbation of viral enhancer II/core promoter.

### Identification of Jmjd3 as a target for miR-939

**Figure 3:**
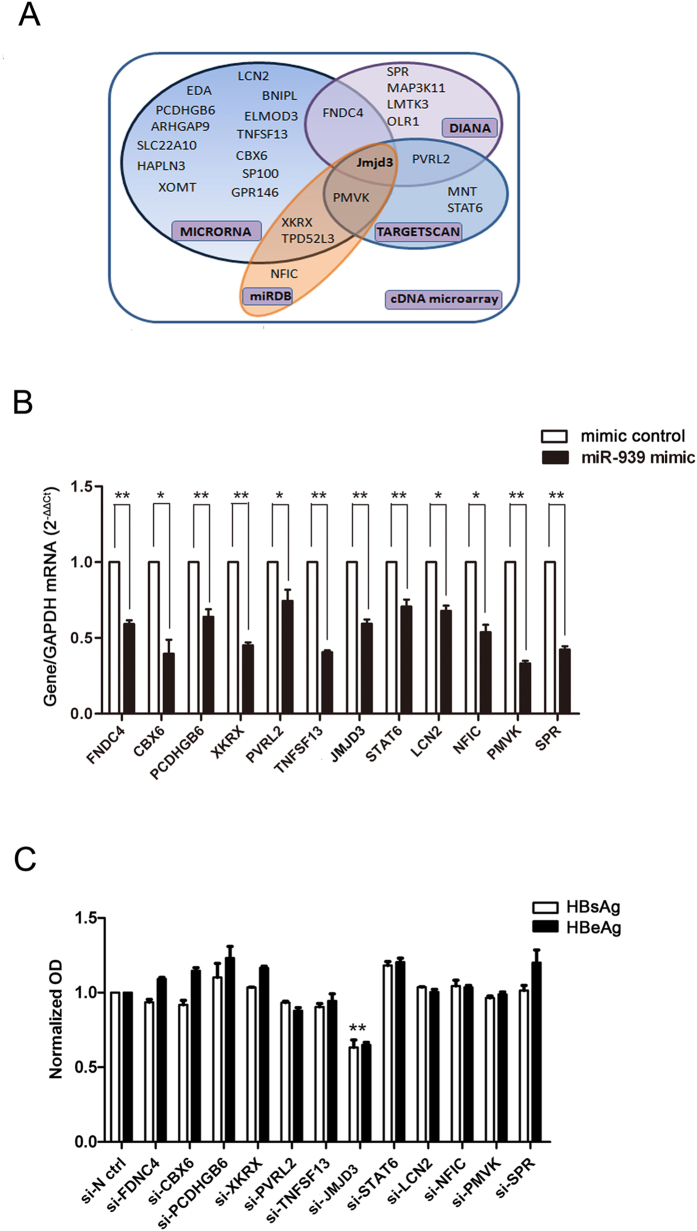
Selection of candidate target genes of miR-939. (**A**) 26 potential target genes resulted from miRNA-databases filteration of differentially expressed genes in cDNA microarray. (**B**) 100 nM indicated miR-939 mimic or mimic control were transfected into Huh7 cells. RNA was isolated and assayed by quantitative RT-PCR analysis using primers specific for each of the 12 candidate miRNAs and GAPDH. Results are presented relative to the value of GAPDH (2^−ΔΔCt^). (**C**) The siRNAs specific for each of the 12 candidate miRNAs were separately co-transfected with pHBV1.3 into Huh7 cells for 48 h. The levels of HBsAg and HBeAg in the supernatant were detected by ELISA. *P < 0.05, **P < 0.01.

Many host factors are reported to bind to HBV enhancer II/core promoter and play important roles in regulating its transcriptional activity. The above results suggested that miR-939 may target host factors that are important regulators of viral transcription. To search for the cellular target of miR-939, we performed expression profiling of Huh7 cells after introduction of miR-939 using cDNA microarray. A total of 673 genes had an over 2 fold decrease in mRNA level. The differential gene expression was further filtered with the miRNA database which integrates the algorithms from DIANA^20^, MICRORNA^21^, TARGETSCAN^22^, and MIRDB^23^. Collectively, these approaches led to the selection of 26 potential targets (Fig. 3A). Following verification by qRT-PCR, 12 candidate genes were confirmed as targets of miR-939 (Fig. 3B). To determine the specific functional target of miR-939 that affects HBV regulation, the siRNAs for candidate targets were co-transfected with pHBV1.3 into Huh7 cells followed by measurement of secreted HBsAg/HBeAg. The knock down efficiency of each siRNA was confirmed by qRT-PCR (Fig. S3). Interestingly, only the reduction of Jmjd3 expression, predicted by nearly every algorithm as miR-939’s target, could mimic the effect of miR-939 (Fig. 3C). Knock-down of Jmjd3 had no significant effect on the expression of albumin or other cellular proteins (Fig. S4), which ruled out the possibility of non-specific artefacts.

**Figure 4:**
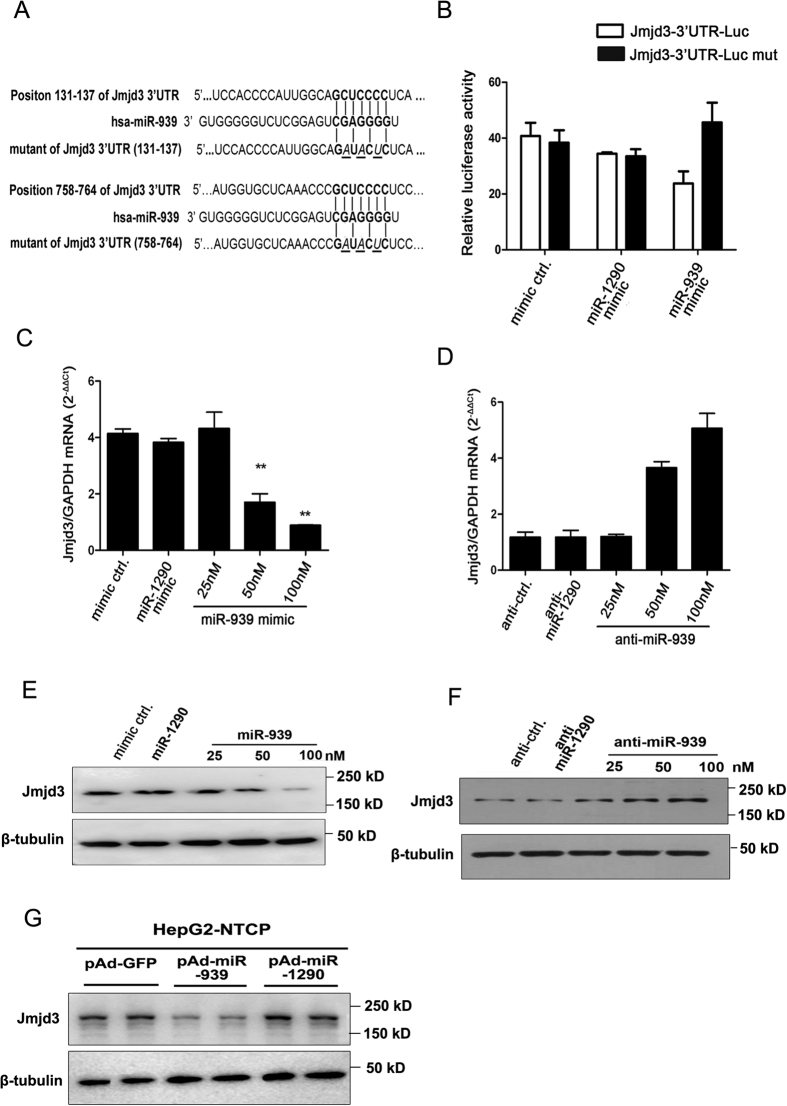
Identification of Jmjd3 as the functional target for miR-939. (**A**) The potential miR-939 binding sites in 3′-UTR of Jmjd3 and the mutant sequences, as predicted by TargetScan 5.1. (**B**) 0.1 μg wild or mutant Jmjd3 3′UTR luciferase reporter plasmid were co-transfected with 100 nM miR-939 mimic or control mimics into Huh7 cells for 48 h, the luciferase activity was then assessed. (**C–F**) Indicated concentration of miR-939 mimics (**C,E**) or inhibitors (**D,F**) were transfected into Huh7 cells. The level of Jmjd3 mRNA (**C,D**) was examined by quantitative RT-PCR analysis. The level of Jmjd3 protein was detected by Western Blotting assay (**E,F**). β-tubulin was detected as a loading control. (**G**) HepG2-NTCP cells were transduced with either control or miR-939-expressing adenoviral vectors. 5 days later, expression of Jmjd3 was detected by Western blotting. Full-length blots are presented in Supplementary Figure S8.

Jmjd3, also known as KDM6B (Lysine (K)-specific demethylase 6B), belongs to JmjC family histone demethylase and has been shown to remove the histone H3 lysine 27 trimethylation (H3K27me3) that is associated with transcriptional repression^24^,^25^. To further examine whether miR-939 could regulate Jmjd3 expression, we analyzed the miR-939 targeting sequence in the 3′UTR of Jmjd3 by TargetScan4.1. Two conserved sites, each with seven nucleotides long, showed complete complementarity to miR-939’s seed region. Next, we generated luciferase reporter constructs containing either the wild-type or mutant Jmjd3 3′UTR sequence (Fig. 4A). The wild or mutant Jmjd3-3′UTR-Luc plasmids were co-transfected with miR-939 mimic or control mimics. The results showed that miR-939 mimic reduced the luciferase activity of wild type Jmjd3-3′UTR-Luc by about 50%, whereas mimic control or miR-1290 mimic had no effect on luciferase activities (Fig. 4B). Moreover, miR-939 mimic had no inhibitory effect on the mutant Jmjd3-3′UTR-Luc (Fig. 4B). Further, we transfected Huh7 cell with increasing doses of miR-939 mimic and measured the levels of Jmjd3 mRNA and proteins. The result showed that expression of miRNA-939 led to inhibition of both the mRNA and protein levels of Jmjd3 in a dose dependent manner (Fig. 4C,E). On the other hand, depletion of miR-939 by its antagomir led to a dose-dependent increase of Jmjd3 mRNA and protein levels (Fig. 4D,F). We also confirmed the effect of miR-939 on Jmjd3 expression in HepG2-NTCP cells with similar findings (Fig. 4G). Thus, these results established that Jmjd3 is a bona-fide target for miR-939.

### Jmjd3 modulates HBV transcription in a demethylase-independent manner

**Figure 5:**
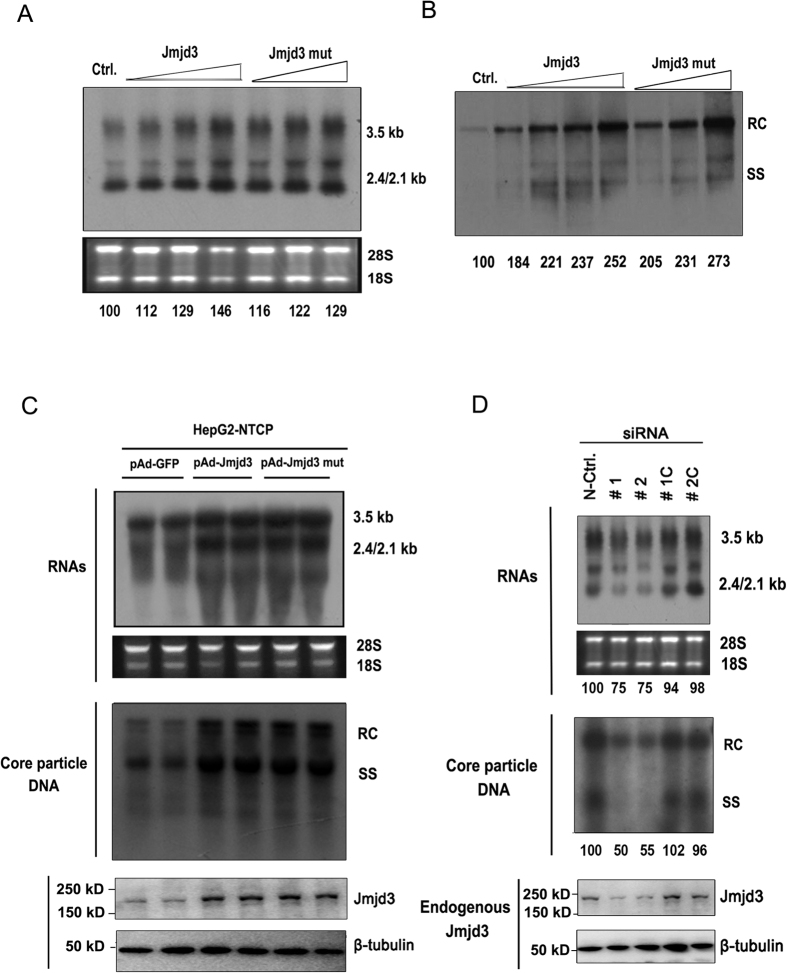
Jmjd3 modulates HBV replication in a demethylase-independent manner. (**A,B**) 0.1 μg pHBV1.3 was co-transfected with either 0.2 μg, 0.4 μg and 0.6 μg wild-type or H1390A mutant Jmjd3 expressing plasmids into Huh7 cells. The HBV RNAs and Core particle DNA were analyzed by Northern blot assay (**A**) and Southern blot assay (**B**). (**C**) HepG2-NTCP cells were infected with HBV in duplicate. 2 days later, cells were transduced with either control or miR-939-expressing adenoviral vectors, and then cultured for an additional 5 days. Intracellular viral RNAs and Core particle DNA were analyzed by Northern blotting (top) and Southern blotting (middle). Expression of Jmjd3 was shown by Western blotting (bottom). (**D**) Two Jmjd3-specific siRNAs (#1, #2), their seed sequence-matched control siRNAs (#1C, #2C) or irrelevant siRNA (N-ctrl) were separately co-transfected with pHBV1.3 into Huh7 cells. The HBV RNAs and Core particle DNA were analyzed by Northern blot assay (top) and Southern blot assay (middle) separately. The expression levels of Jmjd3 were analyzed by Western blotting (bottom). Full-length gels and blots are presented in Supplementary Figure S9.

Having established Jmjd3 as the functional target of miR-939, we attempted to further explore the exact mechanism by which the miR-939-Jmjd3 axis influences viral transcription. We first asked whether perturbation of Jmjd3 protein level would affect HBV transcription and replication. As shown in Fig. 5A,B, over-expression of Jmjd3 boosted HBV mRNA transcripts and replicative intermediates in a dose dependent manner. Conversely, down-regulation of Jmjd3 by RNAi led to pronounced decrease of HBV transcripts and viral replicative intermediates (Fig. 5D). To determine whether the histone H3K27me3 demethylase was required for Jmjd3-mediated increase of HBV replication, we used a demethylase defective Jmjd3 mutant which contained point mutation in the active site of the catalytic domain (H1390A)^24^,^26^. The dysfunction of Jmjd3 H1390A mutant was confirmed by checking the KRT1 RNA in HaCAT cells (Fig. S5)^26^. Huh7 cells were separately co-transfected with either wild-type or mutant Jmjd3 along with pHBV1.3. Unexpectedly, ectopic expression of Jmjd3 mutant yielded the same changes in the levels of HBV mRNA transcripts and replicative intermediates as the wild-type Jmjd3 did (Fig. 5A,B). In HepG2-NTCP infection system, we also observed the similar results (Fig. 5C). These results suggested that the demethylase activity of Jmjd3 was not required for modulating HBV transcription and replication.

### The miR-939-Jmjd3 axis affects C/EBPα dependent HBV enhancer II/core promoter transcription

**Figure 6:**
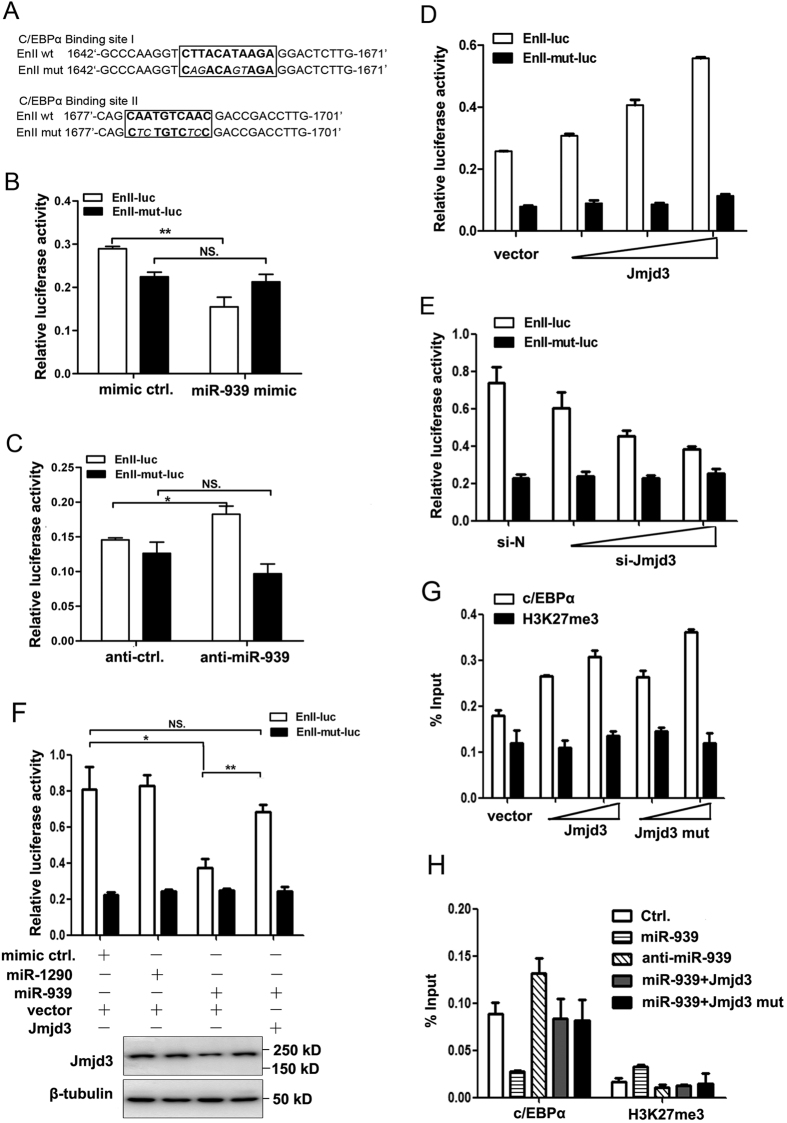
The miR-939-Jmjd3 axis affects C/EBPα dependent HBV enhancer II/core promoter transcription. The mutant sequences of the two important C/EBPα binding sites on En II. (**B,C**) Wild-type or mutant En II reporter plasmid was co-transfected with miR-939 mimic (**B**) or inhibitor (**C**) into Huh7 cells for 48 h, the luciferase activity was then assessed. (**D,E**) Huh7 cells were co-transfected with wild-type or mutant En II reporter plasmid and Jmjd3 expression plasmids (**D**) or si-Jmjd3 (#1), the luciferase activity was then assessed. (**F**) Wild-type or mutant En II promoter reporter plasmid was co-transfected into Huh7 cells as indicated, the luciferase activity was then assessed. The expression of Jmjd3 was detected by Western blotting. Full-length blots are presented in Supplementary Figure S11. (**G**) Huh7 cells were co transfected with wild-type or mutant Jmjd3 expression plasmid. ChIP was performed by using specific antibodies to C/EBPα, H3K27me3 or a non-specific IgG antibody control. Specific primers were used to amplify selectively the HBV En II sequence. Samples were normalized to an aliquot of the total input that was also amplified with gene specific primers to HBV enhancer II/core promoter region. (**H**) HepG2-NTCP cells were transduced with indicated viral vectors. 2 days later, cells were infected with HBV and then cultured for an additional 7 days. Cells were harvested for ChIP assays.

Based on the comprehensive literatures on regulation of HBV En II, C/EBPα was found to be the most important transcription factor. Five conserved c/EBPα binding sites in the sequence of En II were reported. Among them, two have been shown to play the major role in promoting HBV transcription activity^27^. We hypothesized that miR-939 affects En II activity via C/EBPα in a Jmjd3-dependent manner. To test this hypothesis, we constructed a reporter plasmid, which contained the En II sequence with the two most important C/EBPα binding sites mutated (Fig. 6A). ChIP assay confirmed that C/EBPα bound more efficiently to the wild-type En II reporter plasmid than to the mutant (Fig. S6A), and C/EBPα could dose-dependently increase the activity of the wild-type plasmid, but not the mutant (Fig. S6B). As a result, miR-939 failed to suppress the transcription activity of the mutant En II reporter plasmid while the construct containing wild-type En II was fully susceptible to miR-939-mediated suppression (Fig. 6B). On the other hand, transfection of Huh7 cells with miR-939 antagomir led to increased luciferase activity of the reporter containing the wild-type En II but had no effect on the mutant En II (Fig. 6C). Moreover, Jmjd3 over-expression increased the transcription activity of the wild-type En II (Fig. 6D) while down-regulation of Jmjd3 had an opposite effect (Fig. 6E). In comparison, the mutant reporter lost responsiveness to either treatment (Fig. 6D,E). The functional link between miR-939-Jmjd3 axis and C/EBPα was further tested with rescue experiments. Over-expression of Jmjd3, whose mRNA does not contain miR-939 susceptible 3′UTR, reversed the inhibitory effect of miR-939 on En II (Fig. 6F). However, when the C/EBPα binding sites were mutated, the reporter expression lost responses to both miR-939 and Jmjd3 (Fig. 6F). Thus, we concluded that miR-939-mediated suppression of Jmjd3 led to a diminished transcription of HBV En II promoter, which required C/EBPα participation. Based on these observations, we further examined, employing the ChIP assay, whether the physical occupancy of C/EBPα on HBV promoter was indeed regulated by Jmjd3. As expected, overexpression of Jmjd3 led to increased binding of C/EBPα to En II in a dose dependent manner (Fig. 6G). In addition, the demethylase mutant of Jmjd3 exhibited similar effects (Fig. 6G), which indicated Jmjd3 induced recruitment of c/EBPα to the HBV En II was also demethylase independent. We also detected the H3K27me3 levels on HBV En II promoter after introduction of Jmjd3. Although over-expression of Jmjd3 led to increased binding of C/EBPα to En II, the Jmjd3 didn’t change the H3K27me3 levels on HBV En II (Fig. 6G). We repeated the ChIP assays in HepG2-NTCP infection system and obtained similar results supporting our hypothesis (Fig. 6H). Collectively, the above results established that the miR-939-Jmjd3 axis perturbed C/EBPα occupancy on the HBV Enhancer II/core promoter which in turn influenced viral transcription.

### Jmjd3 regulates the physical interaction between C/EBPα and Brm-containing SWI/SNF complex

**Figure 7:**
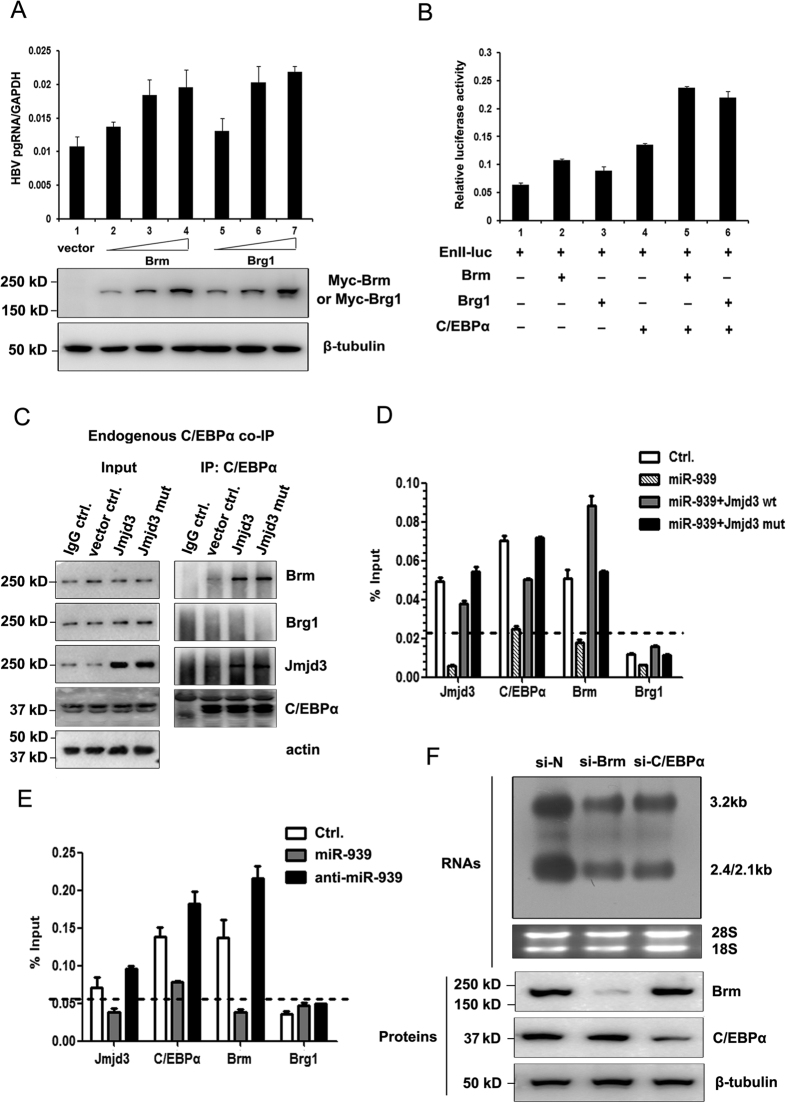
Jmjd3 regulates the physical interaction between C/EBPα and Brm-containing SWI/SNF complex. (**A**) 0.1 μg pHBV1.3 was co-transfected with 0.1 μg, 0.2 μg and 0.4 μg Brm or Brg1-myc expressing plasmids into Huh7 cells. HBV pgRNA levels were analysed by quantitative RT-PCR. Expression levels of Brm and Brg1-myc were analyzed by Western blotting. (**B**) Huh7 cells were transfected as indicated. The luciferase activity was then assessed. (**C)** Huh7 cells were transfected as indicated above each lane. Whole cell extracts were prepared and immunoprecipitated with antibodies specific to C/EBPα. Immunocomplexes were resolved by SDS page and Western blot analysis with specific antibodies as indicated to the right of the figure was performed. (**D**) Huh7 cells were transfected as indicated and ChIP assay was performed with antibodies specific for C/EBPα, Brm, Brg1, Jmjd3, or control IgG. The non-specific IgG control signal was presented as dash line. (**E**) HepG2-NTCP cells were transduced with indicated viral vectors. 2 days later, cells were infected with HBV and then cultured for an additional 7 days. Cells were harvested for ChIP assays. **(F**) HepG2-NTCP cells were transduced with indicated siRNA-expressing lentiviral vectors. 2 days later, cells were infected with HBV and then cultured for an additional 7 days. The HBV RNAs were analyzed by Northern blot assay (top) and the expressions of indicated proteins were analyzed by Western blotting (bottom). Full-length blots are presented in Supplementary Figure S10.

The demethylase-independent activity of Jmjd3 implied that Jmjd3 may not affect the HBV enhancer II transcription by altering histone methylation. This prompted us to find alternative explanations. Several reports suggested that Jmjd3 plays a role in general chromatin remodeling^28^ via interaction with an ATPase subunit of the SWI/SNF chromatin remodeling complex, Brg1^28^,^29^ and this interaction occurs in histone demethylase-independent manner. Moreover, the interaction between C/EBPα and SWI/SNF complex has also been described^30^,^31^,^32^,^33^. Indeed, we found that both Brm and Brg1 exhibited a promoting effect on HBV transcription (Fig. 7A) and En II promoter activity (Fig. 7B). Furthermore, Brm and Brg1 could both enhance the C/EBPa-mediated transcription activity (Fig. 7B). Thus, we speculated that Jmjd3 may regulate HBV transcription by facilitating the recruitment and docking of C/EBPα and SWI/SNF complex on the HBV Enhancer II/core promoter. To explore this possibility, we first performed co-immunoprecipitation (Co-IP) experiments to determine if endogenous C/EBPα could form a complex with Jmjd3 and SWI/SNF subunit, Brg1 or Brm. As shown in Fig. 7C, a clear co-precipitation of C/EBPα with Jmjd3 and Brm, but not Brg1, was observed in Jmjd3 over-expressing Huh7 cells. Importantly, the mutant Jmjd3 could interact with C/EBPα as the wild-type counterpart. Likewise, the binding between Brm and C/EBPα was also maintained in the presence of demethylase mutated Jmjd3. These results suggested that Jmjd3 facilitated the formation of a transcriptional cluster composed of C/EBPα and Brm crucial for synthesis of viral RNAs. Next, we asked whether the physical occupancy of C/EBPα, Jmjd3 and Brm on viral enhancer II/core promoter was altered in the presence of miR-939. Using the ChIP assay, we detected the binding of Jmjd3, C/EBPα and Brm, but not Brg1, to HBV enhancer II/core promoter. As expected, down-regulation of Jmjd3 by miR-939 decreased the binding of C/EBPα and Brm to the HBV enhancer II/core promoter to a level similar to IgG control (Fig. 7D). In addition, the effect of miR-939 could be rescued by both wild-type and mutant Jmjd3 (Fig. 7D). The regulation of miR-939 on C/EBPα and Brm binding to the HBV enhancer II/core promoter was confirmed in HepG2-NTCP infection system (Fig. 7E). Furthermore, down regulation of C/EBPα or Brm led to a decreased level of viral transcripts (Fig. 7F). These results further supported the notion that miR-939 mediated anti-HBV effect could be explained by dysregulated transcriptional machinery composed of Jmjd3, C/EBPα and Brm-containing SWI/SNF complex on HBV Enhancer II/core promoter.

## Discussion

A growing body of evidence has shown that cellular miRNAs have the potential to either directly target HBV viral transcripts, or modulate viral transcription and replication by modifying host gene expression^34^. In this study, we investigated the mechanism by which miR-939 regulates HBV life cycle and demonstrated that miR-939 inhibits viral replication at the level of viral transcription. We confirmed the inhibitory effect of miR-939 on HBV transcription and replication in either transient transfection system or HBV infection system, where the templates for viral transcription were natural episomal cccDNA. Earlier reports showing a robust induction of miR-939 in primary human hepatocytes after LPS or cytokine mix stimulation^35^. In addition, Ouda *et al*. noted that RIG-I overexpression can upregulate miR-939^36^. These data indicated that miR-939 is an inflammation inducible gene. Therefore, it is plausible that miR-939 participates in the host defense against HBV in the immune activation phase of chronic hepatitis B infection.

Expression profiling in conjunction with miRNA databases searches and functional validation identified Jmjd3 as a bona fide target for miR-939. Jmjd3, first identified as one of histone H3K27 demethylases in 2007^24^,^37^, is reported to participate in epidermal differentiation^26^ and is required for neural commitment^38^. Later, in primary peripheral macrophages^37^ and microglial^39^ cultures, Jmjd3 was shown to be an important regulator of LPS-induced inflammatory gene transcription. In accordance with its transcription regulatory role, we found that the miR-939-Jmjd3 axis modulated transcription of HBV Enhancer II/core promoter. Moreover, C/EBPα, an important transcription factor on En II, was indispensable for Jmjd3 mediated transcription enhancement. Indeed, ChIP assay revealed increased occupancy of C/EBPα on En II nucleosome after Jmjd3 over-expression. However, it should be noted that there are other transcription factors whose binding sites at HBV EnII promoter might overlapped with C/EBPα^6^, and the C/EBP family members are able to form homodimers or heterodimers for DNA sequence-specific binding^40^. It is possible that other transcription factors such as C/EBPβ and HNF4α, are also involved in the miR-939 induced inhibitory effect on HBV transcription.

Curiously, the intrinsic demethylase activity of Jmjd3 was not required for this process. Indeed, similar observations have been made by De Santa *et al*. Using a genome-wide ChIP-seq method, they found that the majority of Jmjd3 target genes were not associated with detectable level of H3K27me3^41^ suggesting an H3K27 demethylation-independent mechanism. Recently, several studies have reported low levels of H3K27me3 at HBV promoters in HBV infected HepaRG and NTCP-HepG2 cells, primary human hepatocytes, as well as HBV-infected liver tissue^42^,^43^. We also found that in our system, the level of H3K27me3 at the HBV En II promoter was extremely low compared to the promoter of IGF2 gene, whose expression is under control of promoter histone H3K27 methylation (data not shown)^44^. These might explain the phenomenon we observed that overexpression of Jmjd3 did not lead to the change of H3K27me3 levels at HBV En II.

To further explore the mechanism by which Jmjd3 regulates the HBV Enhancer II/core promoter in demethylase-independent manner, we reasoned that Jmjd3 may further recruit other factors important for RNA synthesis. Indeed, Miller *et al*. also found that Jmjd3 is necessary for IFN-γ production in T cells and this function does not require its demethylase activity^28^. Rather, Jmjd3 acquired chromatin remodeling activity for optimal expression of T-box protein-mediated gene expression via recruitment of T-box transcription factor, T-bet and a Brg1-containing SWI/SNF complex. The chromatin remodeling complex SWI/SNF is known to regulate the transcription of genes by controlling chromatin structure in an ATP-dependent manner. Human SWI/SNF complexes, required for a wide variety of transcriptional programs during development, consist of ~15 subunits and contain Brg1 or Brm exclusively as the ATPase subunits^45^,^46^,^47^. Moreover, the physical and functional interaction between C/EBPα and SWI/SNF complexes had also been documented^30^,^32^,^48^. In hepatocytes, the ATPase subunit of SWI/SNF complex, i.e. Brm and Brg1 can be coupled with C/EBPα which are instrumental for induction of the albumin gene expression^31^. Indeed, our co-immunoprecipitation assays confirmed the interactions of Jmjd3 and Brm containing SWI/SNF complex by C/EBPα. Interestingly, Inayoshi *et al*. also reported the dynamic change in the expression level of Brg1 and Brm during murine liver cells differentiation where the expression of Brm gradually increases and that of Brg1 decreases or remains constant^31^,^33^. Brg1 facilitates the expression of albumin in fetal hepatocytes, and Brm plays an important role in adult hepatocytes^31^. This observation provides a reasonable explanation for our results that only the co-precipitation of C/EBPα and Brm was detected.

**Figure 8:**
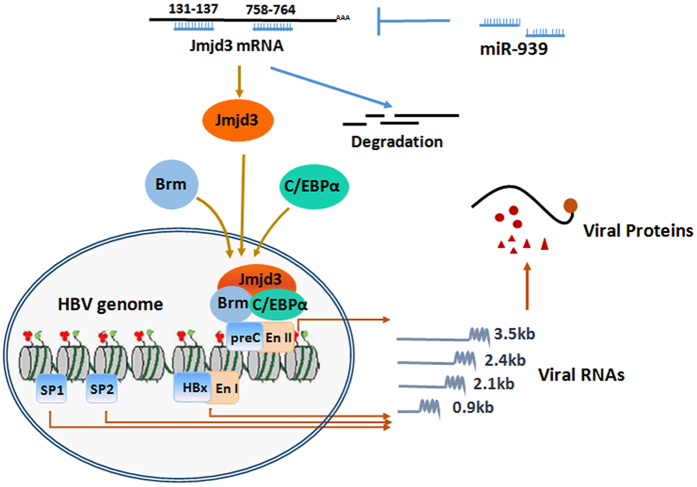
A schematic diagram of the effect of miR-939 on HBV transcription. To facilitate allocation, the HBV genome is linearized. The four HBV promoter and enhancer sites are schematically depicted as boxes.

In conclusion, we uncovered a novel mechanism by which miR-939 interferes with HBV replication, by targeting Jmjd3-mediated docking of chromatin remodeling factors and transcription factors on the HBV Enhancer II/core promoter. Curiously, the intrinsic demethylase activity of Jmjd3 was not required in this process. Figure 8 depicts our working model showing the effect of miR-939 on HBV transcription. It is notable that miR-939 might alter multiple cellular processes that can regulate HBV transcription and the physiological significance of miR-939’s antiviral effects *in vivo* awaits further verification. These results expanded our knowledge on the multi-layered virus-host interactions during the progression of chronic hepatitis B infection. Further endeavors to sagaciously evaluate the significance of all these miRNA-HBV interactions^14^ will hopefully yield feasible therapeutic targets for an ultimate cure of HBV.

## Methods

### Plasmids and Reagents

Plasmid pHBV1.3 encoded a 1.3-mer overlength HBV genome (subtype adw; GenBank accession no. AF100309)^49^. The four HBV promoter/enhancers reporter plasmids were created by inserting the artificial promoter and HBV regulatory elements (Sp1, nt 2704–2823; Sp2, nt 2978–3207; ENI/Xp, nt 957–1354; ENII/Cp, nt 1627–1878) into pGL2basic (Promega)^50^. The luciferase reporter constructs of HBV pgRNA fragments were described previously^18^. To construct Jmjd3-3′UTR-luc, the 3′UTR of Jmjd3 were inserted into the SpeI and HindIII sites of pMIR-REPORT miRNA Expression Reporter (Thermo). The Jmjd3-3′UTR-mut-luc and C/EBPα-EnII-mut-luc were generated by point mutations in Jmjd3 3′UTR or HBV ENII/Cp. miRNA mimics, antagomirs and siRNAs were purchased from RiboBio (Guangzhou, China). Anti-Jmjd3 (ab85392) antibodie was purchased from Abcam; anti-Brg1 (sc-10768X), anti-Brm (sc-6450X), anti-C/EBPα (sc-9314X) were all obtained from Santa Cruz.

### Cell Culture and viruses

Human hepatoma cell line Huh7, HepG2-NTCP cells stably expressing human NTCP (kindly provided by Prof. Stephan Urban)^51^ and HepAD38 cells for producing HBV inoculum^52^ were maintained in Dulbecco’s modified Eagle’s medium supplemented with 10% fetal bovine serum (FBS), 100 U/mL penicillin, and 100 μg/mL streptomycin and maintained at 37 °C in 5% CO_2_. HBV inoculum was prepared from the culture media of HepAD38 cells. Briefly, media were cleared through a 0.45 μm filter and concentrated by Amicon^®^ Ultra-15 (Millipore) at 100-fold concentration. The HBV DNA was quantified by careHBV PCR Assay V3 (Qiagen, Shenzhen, China). pAdPLDest-miR-939 and pAdPLDest-Jmjd3 plasmids were constructed by cloning the U6 promoter and miR-939 sequences or Jmjd3 sequences into pAdPLDest vector. pAdPLDest-miR-939 and pAdPLDest-Jmjd3 were degisted by PacI and then transfected into HEK293 cells to produce pAd-miR-939 and pAd-Jmjd3 adenoviruses. pAd-miR-1290, pAd-Jmjd3-mut and pAd-GFP were obtained by the same approach. All the adenoviruses were titrated by TCID50. Lentiviruses pLKO.1-si-Brm and pLKO.1-si-C/EBPα were produced from HEK293T cells by a transfection procedure.

### Infection, transduction and Transfection

HBV infection of HepG2-NTCP cells was performed according to Prof. Stephan Urban’s protocol with slight modification^51^. Briefly, HepG2-NTCP cells were seeded in 6-well plates and infected with HBV at 100 genome equivalent (GEq)/cell in the present of 4% polyethylene glycol (PEG 8000; Sigma) for 16 h, and then rinsed three times with phosphate-buffered saline (PBS) and maintained in the replication medium containing 2.5% dimethyl sulfoxide (DMSO). Adenoviral and Lentiviral transduction were performed by incubation with 10 multiplicity of infection (MOI) of virus for 16 h. HBV constructs permitting virus replication (pHBV1.3), miRNAs, and small interfering RNAs (siRNAs) were transfected into cells using Lipofectamine 2000 (Life technologies) according to the manufacturer’s instructions. Gaussia luciferase expression plasmid was used to monitor the transfection efficiency.

### RNA extraction, RT-PCR and microarray analysis

Total RNA from Huh7 cells or liver biopsies was isolated using TRIzol reagent (Life technologies) followed by standard phenol chloroform extraction procedure. The amount and quality of RNA were measured by Nanodrop. For quantification of mRNAs, one microgram of total RNA was first reverse transcribed using the ReverTra transcription kit followed by quantitative PCR using Thunderbird SYBR qPCR mastermix (both from Toyobo, Osaka, Japan). MicroRNAs were reverse transcribed using Bulge-loop RT primer and subsequently quantified with miRNA qPCR primer sets (both from RiboBio) using miR-U6 as an internal control. To identify target transcript of miRNA-939, Huh7 cells were transfected with miR-939 mimics (100 nM) for 36 h and total RNA was harvested. cDNA microarray was performed using human LncRNA V2.0 from Arraystar Inc, which contains probes for protein coding transcripts and long non-coding RNAs, following the recommended workflow. Differentially expressed genes with over 2 fold change were selected for further filtering and qRT-PCR validation.

### Analysis of HBV Replication and Southern/Northern blotting

HBV replicative intermediates from intracellular core particles and HBV transcripts were extracted from transfected cells according to the published protocols^18^. HBV DNA and RNA were detected by southern/northern blot using PCR-amplified digoxingenin labeled probes (PCR DIG Probe Synthesis Kit, Roche). Briefly, extracted DNA was loaded on 1.2% agarose gel. Total RNA was loaded on formaldehyde denaturing agarose gel. After electrophoresis, DNA/RNA was transferred onto positive charged nylon membrane (Roche) using a vacuum blotter (Bio-rad, USA). Subsequent procedures were performed according to the instruction provided by the DIG High Prime DNA Labeling and Detection Starter Kit II manual (Roche). The levels of HBsAg and HBeAg in culture supernatants were examined by enzyme-linked immunosorbent assay (ELISA) kits (Kehua, Shanghai, China).

### Dual-luciferase reporter assay

Huh7 cells were seeded in a 48-well plate (3 × 10^4^ per well) and cultured overnight. The cells were then co-transfected with 0.1 μg of the lucifearse reporter plasmid, 0.01 μg pRL-TK (expressing Renilla luciferase; Promega) and the indicated amounts of miRNAs or expression plasmids. 48 h later, cells were lysed with passive lysis buffer and assayed for luciferase activity with a Dual-Luciferase Assay kit (Promega) according to the manufacturer’s instructions. Firefly luciferase activities were normalized based on Renilla luciferase activities. All reporter assays were repeated at least three times. The data shown are mean values ± SEM from one representative experiment.

### Immunoprecipitation and Immunoblotting

Cells were harvested in 2 ml lysis buffer (50 mM Tris-HCl [pH 7.4], 350 mM NaCl, 30 mM MgCl_2_, 10 mM EDTA, 0.5% NP-40, 20% glycerol supplemented with Roche protease inhibitors). Collected soluble fraction was first pre-cleared with 1 μg normal IgG and 20 μl protein A/G plus-agarose beads (Santa Cruz) for 2 h, followed by incubation with 5 μg indicated antibodies overnight. The immune complexes were precipitated, washed and prepared in SDS sample buffer. Samples were loaded onto the gel, separated by SDS-PAGE and transferred onto a nitrocellulose membrane (Roche) for Western blotting. After blocking, membranes were cut according to the prestained protein ladder (Biorad, 161-0374) and incubated with indicated primary and corresponding secondary antibodies. Protein bands were visualized using an immobilon western chemiluminescent HRP substrate (Millipore) and observed with the Odyssey Fc imaging system (LI-COR, USA).

### Chromatin Immunoprecipitation (ChIP) Assays

ChIP assays were performed according to Levrero’s protocol^53^ with minor modifications. Briefly, 48 h after transfection, Huh7 cells were resuspended in ChIP lysis buffer and incubated 10 min on ice. The lysate was centrifuged to pellet the nuclei. The supernatant was removed, and the nuclei were fixed in 1% formaldehyde for 30 min on ice. Isolated cross-linked nuclei were sheared by sonication in a 1% SDS lysis buffer to generate chromatin fragments of 200–500 bp. After centrifugation, the supernatant was diluted, pre-cleared with A/G plus agarose beads, and divided into aliquots. 2 × 10^6^ cells were used for one ChIP assay. The chromatin was then subjected to immunoprecipitation for 14–16 h at 4 °C using 5–10 μg indicated antibodies. Immunoprecipitations with normal IgG were included in each experiment as a negative control. The protein/DNA complexes were precipitated with A/G agarose beads and washed. After elution and reverse cross-linking step, chromatin immunoprecipitates were quantified by Taqman PCR amplification (ToYoBo) using the primers specific for the HBV enhancer II/core promoter region. The forward and reverse primers were as follow: 5′-CTCCCCGTCTGTGCCTTCT-3′ and 5′-GCCCCAAAGCCACCCAAG-3′. Hybrodization probe was: 5′-FAM- AGCGAAGTGCACACGGACCGGCAGA-TAM-3′. For samples from HepG2-NTCP infection system, chromatin immunoprecipitates were treated with Plasmid-safe DNase (Epicentre, USA) for 1 h at 37 °C before PCR amplification.

### Equipment and settings

The signals from the Southern blotting and Northern blotting, and the Western blotting of Figs 4F and S4 were visualized on X-ray films, which were then scanned. For western blotting of other Figures, the signals were visualized with the Odyssey Fc imaging system (LI-COR). The protein signals were captured in the Chemi channel and the molecular weight marker signals in the 700 channel. The Adobe Photoshop is used for image process.

### Statistical Analysis

The statistical analysis was performed using PASW 18 and Prism 5 (Graphpad). For cell culture experiments, two-tailed student’s T test was used. A P value less than 0.05 was considered statistically significant. Data from a series of at least three experiments are shown as standard error of the mean (SEM).

## Additional Information

**How to cite this article**: Chen, C. *et al*. MicroRNA-939 restricts Hepatitis B virus by targeting Jmjd3-mediated and C/EBPα-coordinated chromatin remodeling. *Sci. Rep.* **6**, 35974; doi: 10.1038/srep35974 (2016).

## Supplementary Material

Supplementary Information
