# Supplementary material for: MicroRNA-939 restricts Hepatitis B virus by targeting Jmjd3-mediated and C/EBPα-coordinated chromatin remodeling
Source: Sci Rep. 2016 Oct 25;6:35974. doi: 10.1038/srep35974 (PMC5078794; doi:10.1038/srep35974)
Supplement: Supplementary Information [file srep35974-s1.doc]

**MicroRNA-939 restricts Hepatitis B virus by targeting Jmjd3-mediated and C/EBPα-coordinated chromatin remodeling**

**Cuncun Chen^1,3^+, Min Wu^2^+, Wen Zhang^1,3^, Wei Lu^4^, Min Zhang^5^, Zhanqing Zhang^4^, Xiaonan Zhang^2^*, Zhenghong Yuan^1,3^***

**Supplementary information**


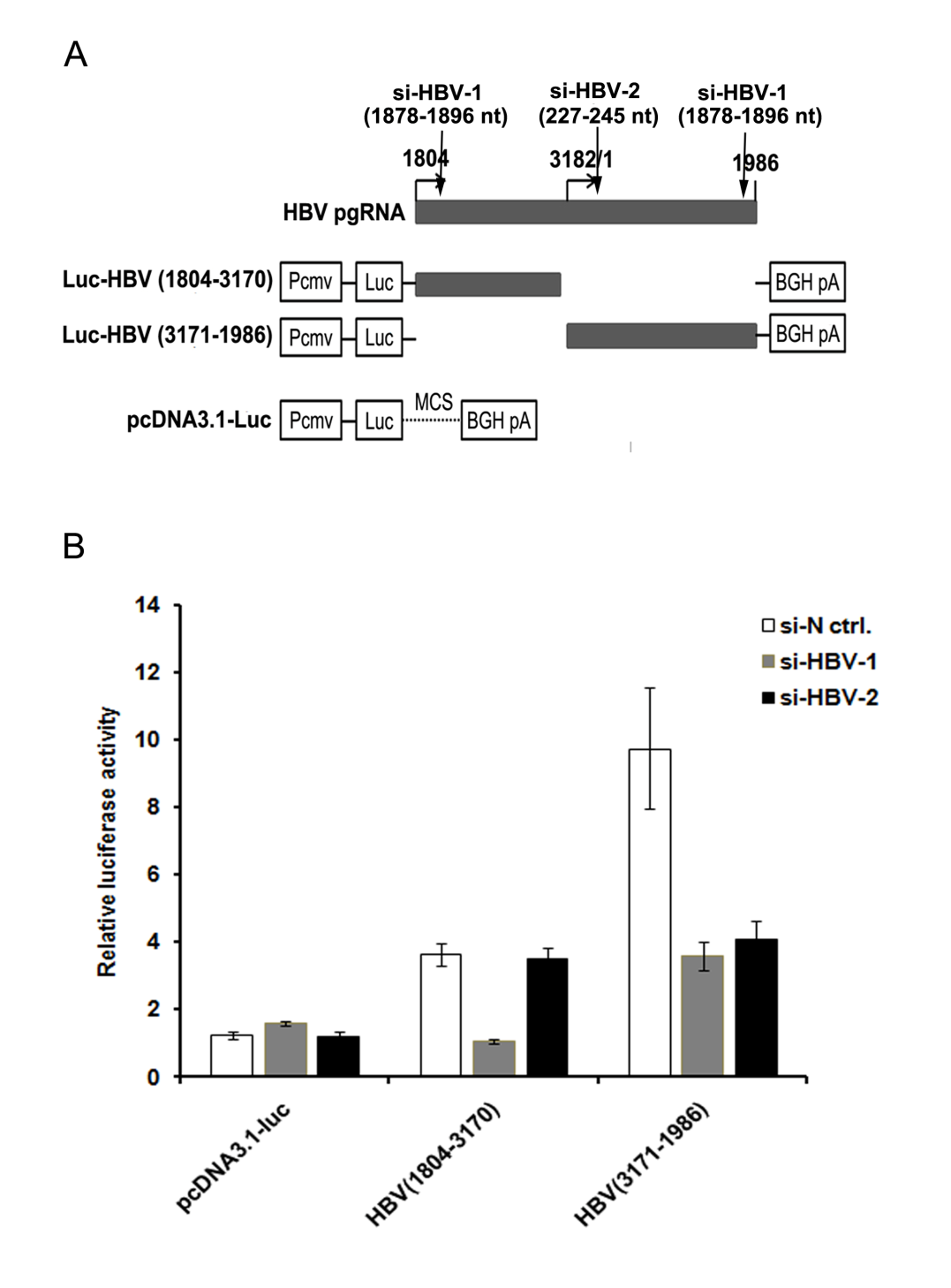
**Fig. S1. Inhibition of chimeric HBV pgRNA luciferase reporter plasmid activities by siRNAs targeting HBV pgRNA sequences**

1. Schematic diagram of the chimeric HBV pgRNA luciferase reporter plasmids and the targeting regions of designed siRNAs. (B) Huh7 cells were transfected with 0.1 μg indicated luciferase fusion constructs and 100 nM si-HBV-1, si-HBV-2 or irrelevant siRNA control separately for 48 h, the luciferase activity was then assessed. Results were normalized according to Renilla luciferase activities.


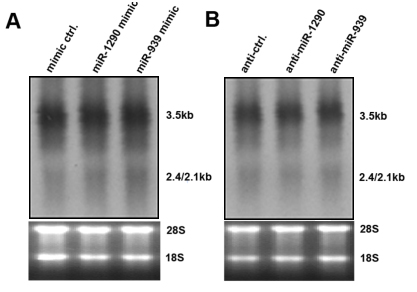
**Fig. S2. miR-939 had no effect on the transcription of pCMV-HBV**

pCMV-HBV plasmid was co-transfected with 50 nM miR-939 mimic (A) or inhibitor (B) into Huh7 cells. miR-1290 was used as an unrelated control. The HBV RNAs were analyzed by Northern blot assay. Full-length gels are presented in Figure S11.

**Fig. S3. The knock down efficiency of 12 siRNAs**

**
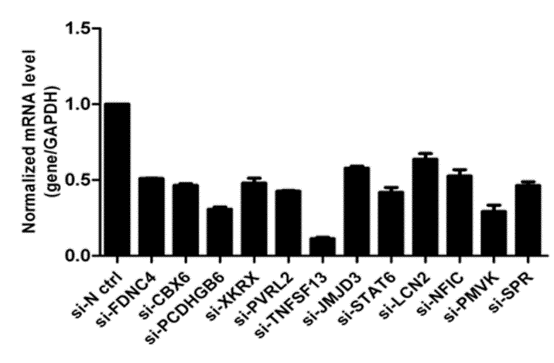
**

mRNA levels of each gene after siRNA knockdown were checked by real-time quantitative RT-PCR.


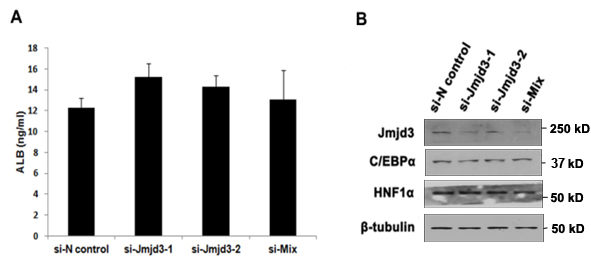
**Fig. S4. Knock-down of Jmjd3 had no significant effect on the expression of albumin or other cellular proteins.**

Huh7 cells were transfected with control siRNA, siJmjd3-1, siJmjd3-2 or mixture for 48 h. (A) The level of albumin in the supernatant were detected by ELISA. (B) The expression levels of indicated proteins were analyzed by Western blot assay. Full-length blots are presented in Figure S11.


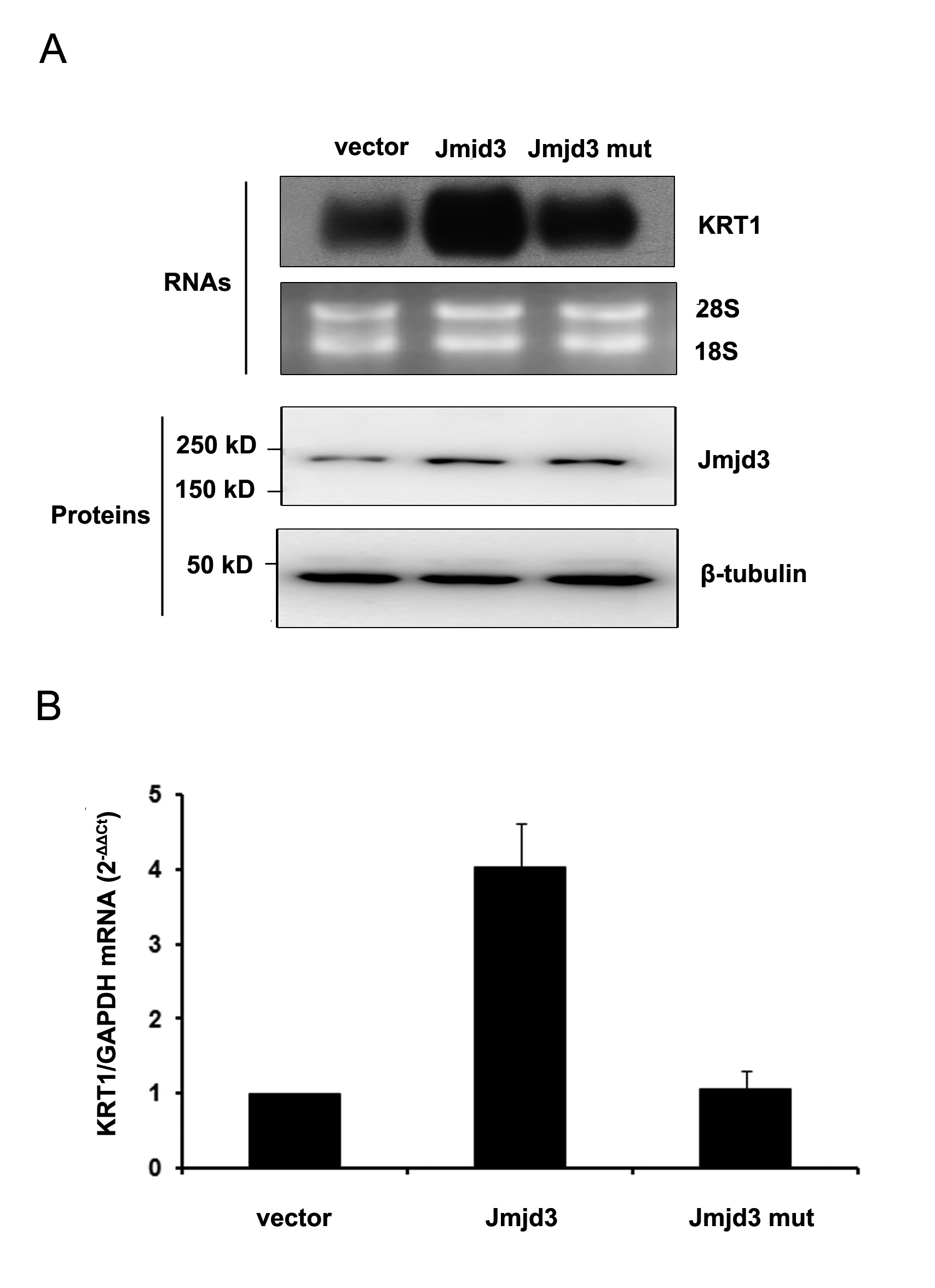
**Fig. S5. Jmjd3, but not Jmjd3 mutant, can induce KRT1 expression**

HaCAT cells were transfected with wild-type or H1390A mutant Jmjd3 expressing plasmids. The KRT1 mRNA was analyzed by Northern blot assay (A) and quantitative RT-PCR (B). Full-length gels and blots are presented in Figure S11.


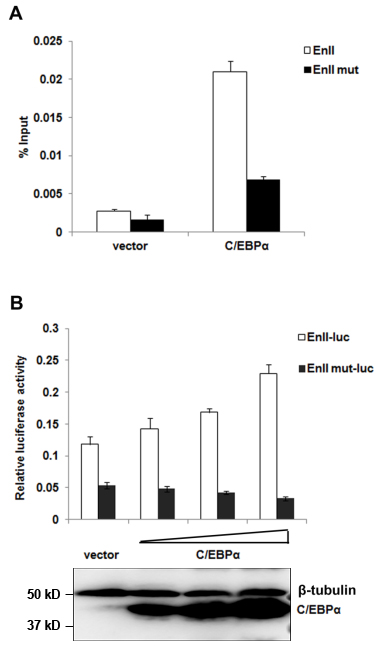
**Fig. S6. Effects of C/EBPα on the wildtype or mutant En II Promoter**

Wild-type or mutant En II reporter plasmid was co-transfected with empty vector or C/EBPα expression plasmid into Huh7 cells for 48 h. ChIP was performed by using specific antibodies to C/EBPα or control IgG. Specific primers were used to amplify the consensus sequence of the wild-type and mutant En II reporter plasmid. (B) Huh7 cells were co-transfected with wild-type or mutant En II reporter plasmid and C/EBPα expression plasmids for 48 h, the luciferase activity was then assessed. Full-length blots are presented in Figure S11.


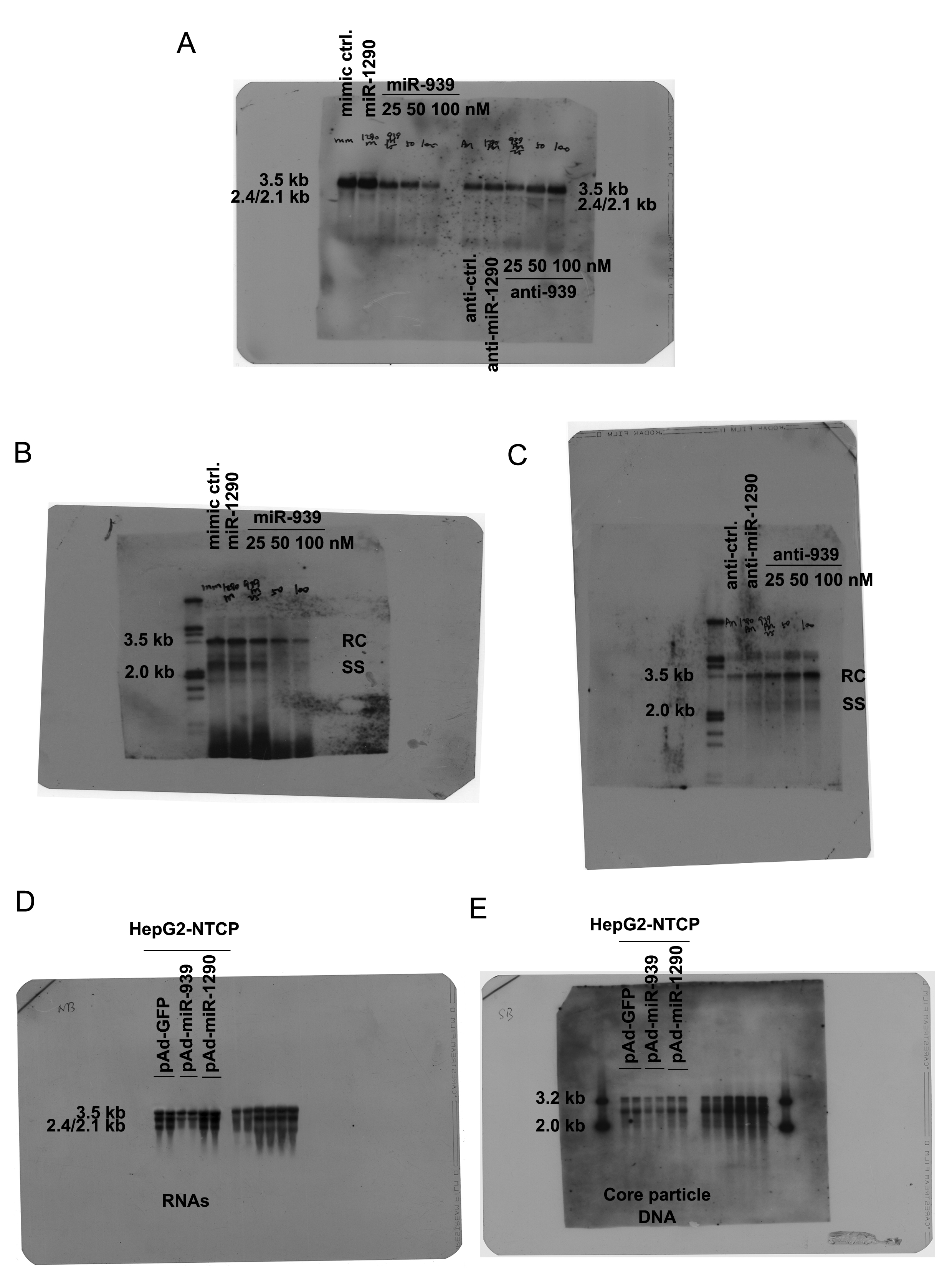
**Fig. S7. Full-length gels of Figure 1**

1. Full-length gels of Fig. 1A (left) and Fig. 1C (right). (B) Full-length gels of Fig. 1B. (C) Full-length gels of Fig. 1D. (D, E) Full-length gels of Fig. 1E. Northern blotting (D) and Southern blotting (E).


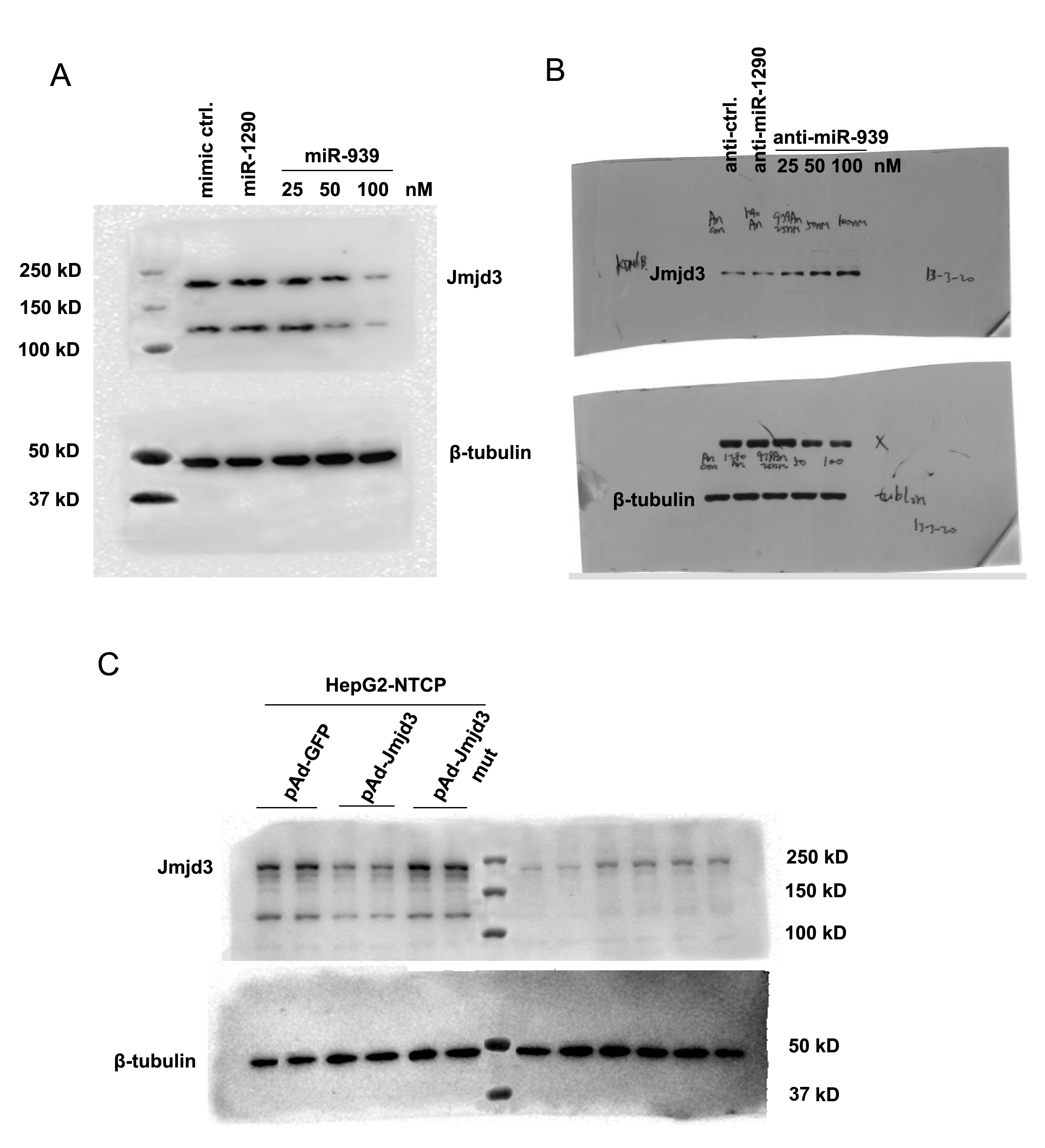
**Fig. S8. Full-length blots of Figure 4**

1. Full-length blots of Fig. 4E. (B) Full-length blots of Fig. 4F. (C) Full-length blots of Fig. 4G.


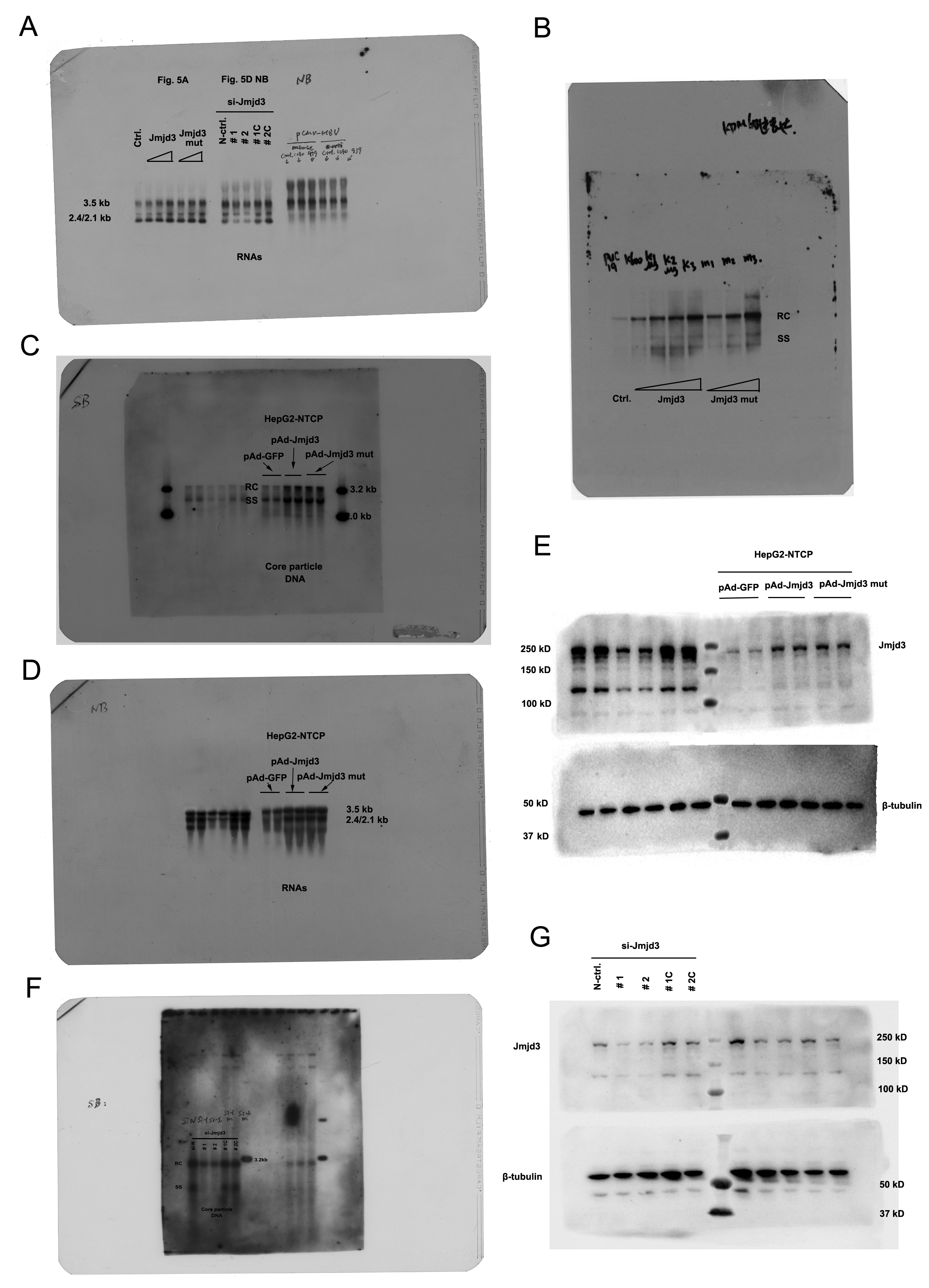
**Fig. S9. Full-length gels and blots of Figure 5**

1. Full-length gels of Fig. 5A (left) and Fig.5D (right). Northern blotting. (B) Full-length gels of Fig. 5B. (C,D,E) Full-length gels and blots of Fig. 5C. Southern blotting (C), Northern blotting (D) and Western blotting (E). (F,G) Full-length gels and blots of Fig. 5D. Southern blotting (F) and Western blotting (G).


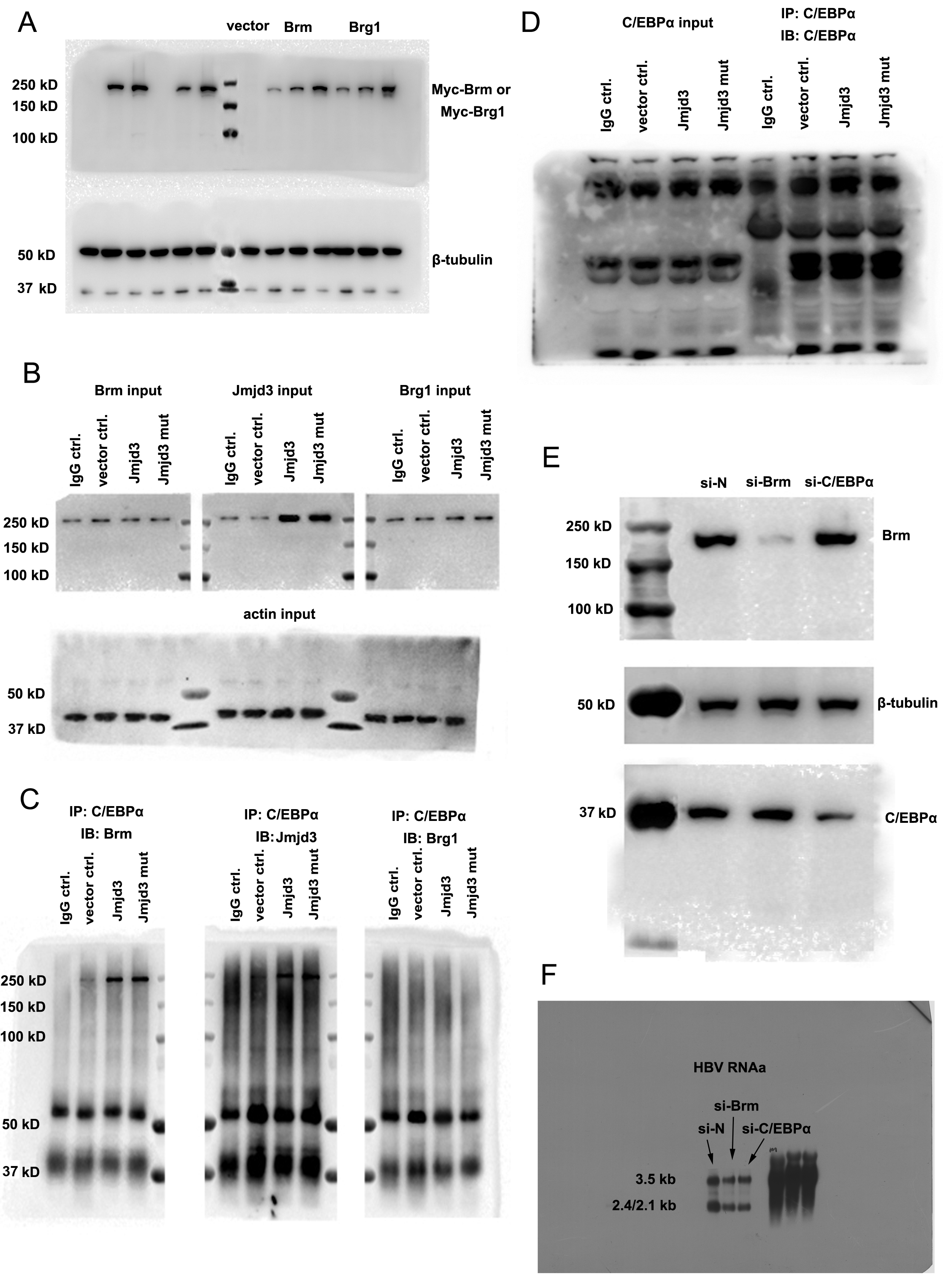
**Fig. S10. Full-length gels and blots of Figure 7**

1. Full-length blots of Fig. 7A. (B,C,D) Full-length blots of Fig. 7C. (E,F) Full-length gels and blots of Fig. 7F.

**Fig. S11. Full-length gels and blots of Figure 4 and Supplementary figures**

**
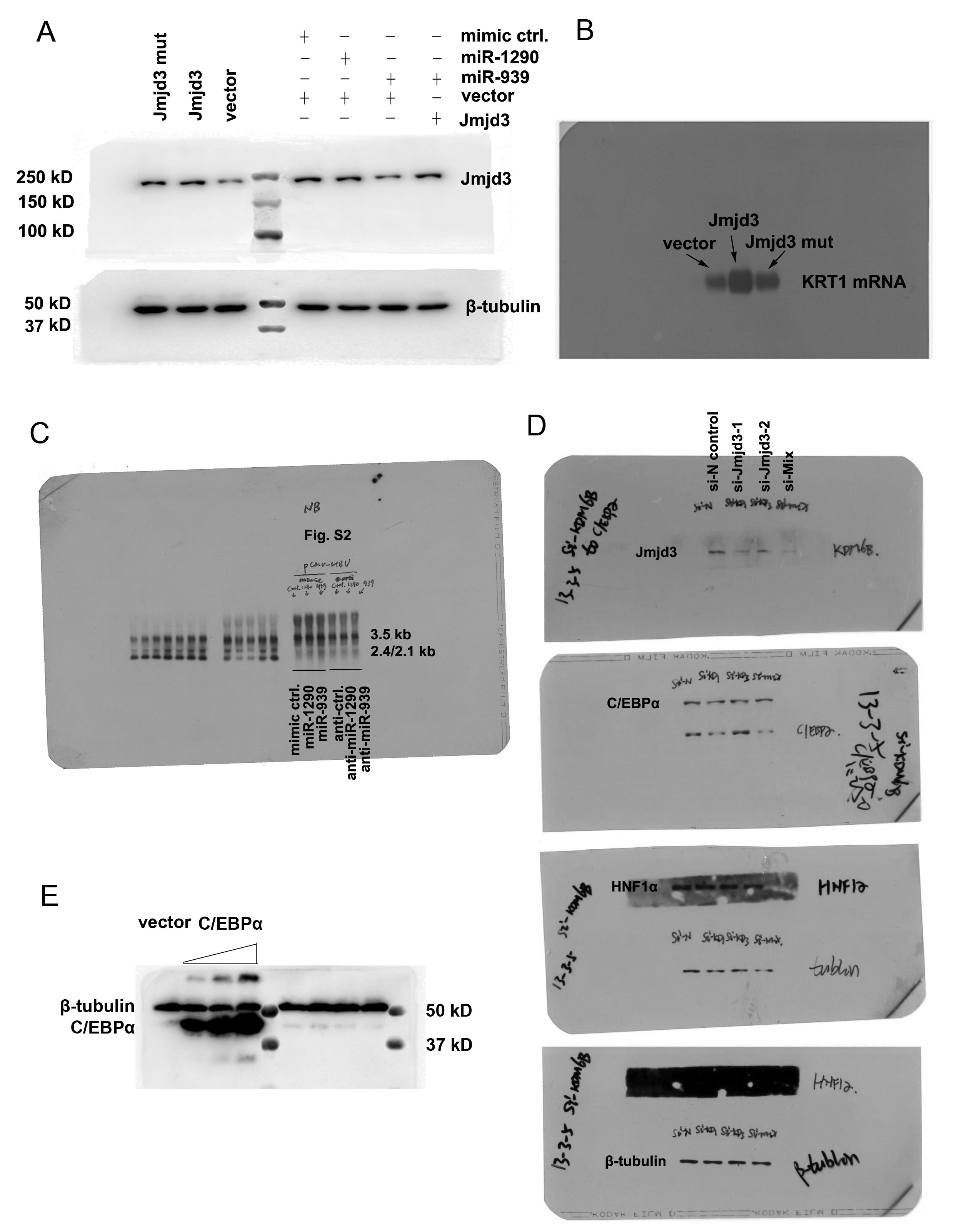
**

1. Full-length blots of Fig. 6F (right) and Fig. S5 (left). (B) Full-length gels of Fig. S5. (C) Full-length gels of Fig. S2. (D) Full-length gels of Fig. S4. (E) Full-length blots of Fig. S6B.

**Supplementary Table 1. Sequences of miRNA, siRNA, and realtime PCR Primers used in this study**

| **miRNA (5’-3’)** | | |
| --- | --- | --- |
| Has-miR-939-mimic (Accession: MIMAT0004982) | | UGGGGAGCUGAGGCUCUGGGGGUG |
| Has-miR-1290 mimic (Accession: MIMAT0005880) | | UGGAUUUUUGGAUCAGGGA |
| miR-939 antagomir | | CACCCCCAGAGCCUCAGCUCCCCA |
| miR-1290 antagomir | | UCCCUGAUCCAAAAAUCCA |
| **Target sequences of siRNA (5’-3’)** | | |
| si-Jmjd3-1 | GCATCTATCTGGAGAGCAA | |
| si-Jmjd3-2 | GATTCTTTCTATGGGCTTT | |
| si-Jmjd3-1-mut | GCATCTATGACGAGAGCAA | |
| si-Jmjd3-2-mut | GATTCTTTGATTGGGCTTT | |
| si-Brm | GCAGGAAACCGAAGAGAAA | |
| si-C/EBPα | GGAGCTGACCAGTGACAAT | |
| si-HBV-1 | GCTGTGCCTTGGGTGGCTT | |
| si-HBV-2 | GAATCCTCACAATACCGCA | |
| **Realtime PCR Primer sequences (5’-3’)** | | |
| HBV EnII | F: CTCCCCGTCTGTGCCTTCT  R: GCCCCAAAGCCACCCAAG  Probe: FAM- AGCGAAGTGCACACGGACCGGCAGA-TAM | |
| Jmjd3 | F: CAGGAGAATAACAACTTC  R: TACACAGGAATATTGGAT | |
| KRT1 | F: GATGTGGATGGTGCTTAT  R: CTTGGTAGAGTGCTGTAA | |
